# Concordance rate of a four-quadrant plot for repeated measurements

**DOI:** 10.1186/s12874-021-01461-0

**Published:** 2021-12-01

**Authors:** Mayu Hiraishi, Kensuke Tanioka, Toshio Shimokawa

**Affiliations:** 1 Clinical Study Support Center, Wakayama Medical University Hospital, Wakayama, Japan; 2 Graduate School of Culture and Information Science, Doshisha University, Kyoto, Japan; 3 Department of Biomedical Sciences and Informatics, Doshisha University, Kyoto, Japan; 4 Department of Medical Data Science, Graduate School of Medicine, Wakayama Medical University, Wakayama, Japan

**Keywords:** Clinical trial, Method comparison, Monte Carlo simulation, Trending agreement

## Abstract

**Background:**

To assure the equivalence between new clinical measurement methods and the standard methods, the four-quadrant plot and the plot’s concordance rate is used in clinical practice, along with Bland-Altman analysis. The conventional concordance rate does not consider the correlation among the data on individual subjects, which may affect its proper evaluation.

**Methods:**

We propose a new concordance rate for the four-quadrant plot based on multivariate normal distribution to take into account the covariance within each individual subject. The proposed concordance rate is formulated as the conditional probability of the agreement. It contains a parameter to set the minimum concordant number between two measurement methods, which is regarded as agreement. This parameter allows flexibility in the interpretation of the results.

**Results:**

Through numerical simulations, the AUC value of the proposed method was 0.967, while that of the conventional concordance rate was 0.938. In the application to a real example, the AUC value of the proposed method was 0.999 and that of the conventional concordance rate was 0.964.

**Conclusion:**

From the results of numerical simulations and a real example, the proposed concordance rate showed better accuracy and higher diagnosability than the conventional approaches.

## Background

### Introduction

New clinical measurements and new technologies such as cardiac output (CO) monitoring continue to be introduced. It is important that these new technologies are verified to ensure their measurement methods are equivalent to those of the standard measurement methods before implementing them in clinical practice. For example, an improved cardiac index (CI) tracking device was compared with a traditional method for CI by transpulmonary thermodilution to assess its reliability in accurately measuring changes in norepinephrine doses during operations (Monnet et al., [14]). In Cox et al.’s [11] study, bioimpedance electrical cardiometry, another experimental measurement device of CI, was examined using continuous pulmonary artery thermoregulatory catheterization as the gold standard before, during, and after cardiac surgery.

Various statistical methods have been proposed to assess the equivalence of the new testing measurement methods with the gold standards (e.g., Carstensen, [7]; Choudhary and Nagaraja, [10]; Choudhary and Nagaraja, [9]). In Altman and Bland [1], and Bland and Altman [4, 5], the Bland-Altman analysis has been proposed to evaluate the accuracy of a new clinical test based on its difference from the gold standard measurement values and the mean of the two tests values. Shieh [19] also proposes a new method for calculating the sample size when conducting the Bland–Altman analysis during clinical trials. The Bland–Altman analysis has also been expanded to cases of repeated measurement (e.g., Bland and Altman, [6]; Zou, [21]) in clinical studies. Asamoto et al. [3] use this analysis method to evaluate the equivalence of the accuracy in a less-invasive continuous CO monitor during two different surgeries. Meanwhile, the Bland–Altman plot cannot describe the trending ability between the two compared measurements, because this analysis does not consider the order of the observed data. If the signs of the true mean of the differences between each measurement methods’ values at one time point and at the subsequent time point are the same, these two clinical methods are regarded as containing the same trending ability. On the other hand, if these signs are different, the two clinical measures have different trending ability. For the evaluation of this trending ability, the four-quadrant plot is used to draw the changes of the measurement results, and the concordance rate (Perrino et al., [17]; Perrino et al., [16]) is accordingly calculated along with the Bland–Altman analysis in the equivalence comparative clinical trials (e.g., Monnet et al., [14]).

The four-quadrant plot and concordance rate focus on the trending ability between each difference of two testing values. In a four-quadrant plot, pairs of each difference of two testing values at sequential time points are plotted. For example, the plot draws the value at the second time point minus the value measured at the first time point, which are both measured by the gold standard on the horizontal axis, while the difference value between the same time points is measured by the experimental method on the vertical axis.

The evaluation of the four-quadrant plot is based on whether the trends for each difference between the new experimental measurement and the gold standard are concordant. When the trends between the two measurements increase or decrease together, these points are regarded as being in agreement (Saugel et al., [18]). The values with small difference are not counted for the concordance rate through the introduction of the “exclusion zone”. The concordance rate in a four-quadrant plot is calculated using the ratio of the number of agreements to all data points. The conventional concordance rate can be also regarded as a conditional probability under the assumption of a binomial distribution, where a conditional event is an event where the difference in measurements values between the time points is not in the exclusion zone. However, this gives the conventional concordance rate difficulty of considering covariance within an individual, despite one subject is commonly measured multiple times in clinical practice. High covariance within an individual may lead to incorrect results in a calculation if the covariance is not considered in the calculation of the concordance rate. In addition, when calculating the concordance rate based on the conditional probability of the binomial distribution, the difference values fell into the exclusion zone is excluded. This reduces the sample size and may affect the estimated concordance rate.

Our study proposes a new concordance rate for the four-quadrant plot based on multivariate normal distribution to take into account the covariance within each individual subject. The proposed method is described as a conditional probability based on a multivariate normal distribution, while the conventional concordance rate is the conditional probability based on a binomial distribution. It means the proposed methods are essentially the same framework, only with different assumptions. In the proposed method, moreover, we can estimate the parameters of the conditional probability with the values including those fell into the exclusion zone, in other words, without reducing the sample size. Therefore, the proposed concordance rate can overcome the difficulties of the conventional concordance rate on correlation and exclusion zone.

Through a numerical simulation and a real example, we prove the superiority of the proposed method compared with the traditional concordance rate in a practical case. This new method can be applied to any number of repeated measurements. In this study, we examine the case of three time points in a numerical simulation. The proposed method also has a parameter to set the minimum concordant number *m* between two measurement methods, which are regarded as being in agreement. For instance, when the parameter *m* is 3 and *T* is 5, where *T* is the number of the differences in measurement values, the concordance rate evaluates the case of more than 3 agreements out of 5 times. This parameter analysis from a clinical perspective. In general, *T*=*m* and the high probability of the concordance are ideal, but the parameter can provide a more detailed interpretation of the degree of agreement by adjusting the parameter *m*. In clinical practice, it is more natural to assess the concordance more than *m* out of *T* in the repeated measurements. Here, the meaning of “agreement” differs from the assessment of the conventional concordance rate. When the conventional concordance rate is applied to the case of repeated measurements, agreement in this sense cannot be assessed. We will show the validitiy on the proposed method through the numerical situations and the real example.

The paper is organized as follows: the conventional concordance rate for the four-quadrant plot is explained in the following paragraph. In Methods, we introduce the new proposed concordance rate and present the case wherein the maximum number of agreements is two, then explain the application of the proposed method to simulations and to a real data of blood pressure. Results section shows the findings of the simulation and the real data. We have the further consideration in Discussion, and conclude this paper in the last section.

### Conventional concordance rate for the four-quadrant plot

This subsection explains the ways to draw the four-quadrant plot and calculate the concordance rate by using the conventional method. The assessment method for the trending agreement of two testing values using the four-quadrant plot was first proposed by Perrino et al. [17]. The four-quadrant plot uses each pair of differences between the values measured by the two clinical methods being compared. Point $x^{*}_{{it}} (i=1,2,\cdots,n;\quad t=1,2,\cdots,(T+1))$ indicates the value of a gold standard for individual subject *i* at time *t*, and $y^{*}_{{it}} (i=1,2,\cdots,n;\quad t=1,2,\cdots,(T+1))$ is the value of the experimental technique. Then, the *t* difference of the values measured by the gold standard is

$$\begin{array}{*{20}l} x_{{it}} = x^{*}_{i(t+1)} - x^{*}_{{it}} \quad (t=1,2,\cdots,T), \end{array} $$

and the *t* difference of the values measured by the experimental technique is

$$\begin{array}{*{20}l} y_{{it}} = y^{*}_{i(t+1)} - y^{*}_{{it}} \quad (t=1,2,\cdots,T). \end{array} $$

**Fig. 1:**
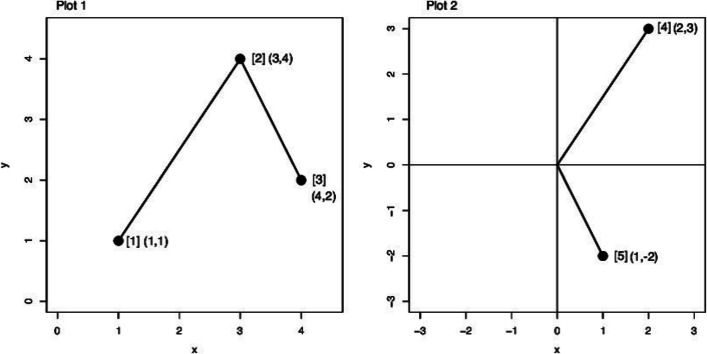
Plots for the step of drawing the four-quadrant plot. The horizontal axis is *x* and the vertical axis is *y*. Plot 1: Data plotted for three pairs of values on Cartesian coordinates. Plot 2: Four-quadrant plot of the data in Plot 1

Plot 1 in Fig. 1 shows an example of the treatment values in a time sequence that compares two tests for one subject. Focusing on the first two data points in Plot 1, the difference between [2] and [1] can be described as [4] of the four-quadrant plot in Plot 2. At this time, both *x* and *y* increase, indicating that the direction of change in *x* and *y* is the same. A point such as [4], plotted in the upper-right of the four-quadrant plot, can be evaluated as being in “agreement.” In contrast, the difference between [3] and [2] is plotted as [5] in the lower-right section of Plot 2. In this case, *x* increases but *y* decreases, that is, the trend of *x* and *y* is recognized as being in “disagreement.” Similarly, if the difference in both *x* and *y* is negative, as plotted in the lower-left, the change is also in “agreement,” while the data points in the upper-left can be assessed as being in “disagreement.”

**Fig. 2:**
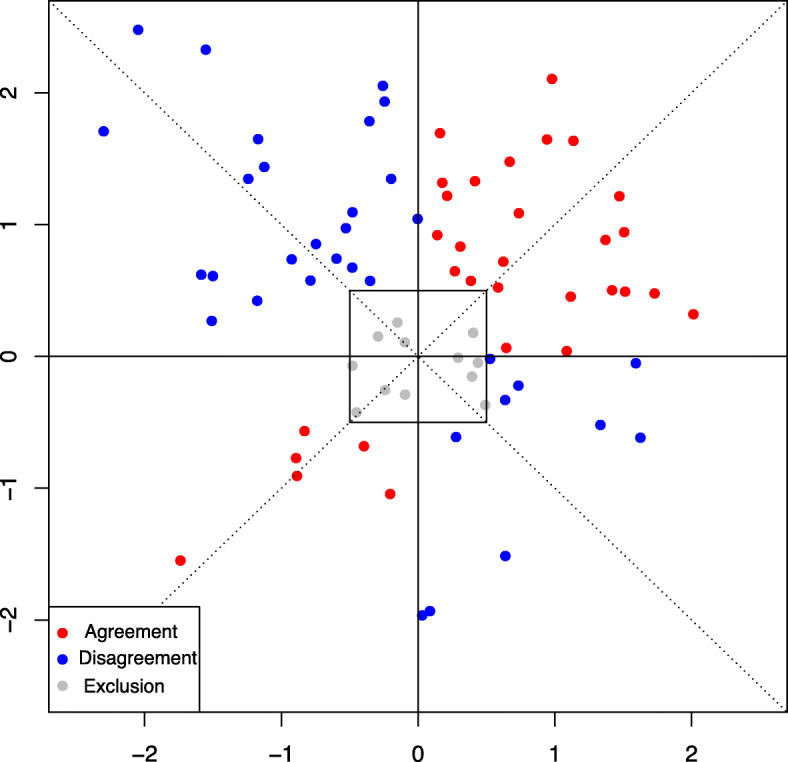
Four-quadrant plot with artificial example data

Figure 2 is a four-quadrant plot with artificial example data. In the figure, the red points in the upper-right and lower-left sections are counted as being in “agreement.” The blue dots, on the other hand, signify “disagreement.” When the difference value of the experimental technique is equal to that of the gold standard, the data dot is on the 45^∘^ line (dotted lines in Fig. 2).

The concordance rate is calculated based on the idea above. The conventional concordance rate (CCR) is defined as follows:

1 $$\begin{array}{*{20}l} \text{CCR}(a) = \frac{ \# \text{SA} - \# \text{AEz}(a) }{ nT - \# \text{Ez} (a) }, \end{array} $$

where

$$\begin{aligned} \text{SA} =& \{(x_{{it}}, y_{{it}})| \; {\big(} (x_{{it}}\geq0,\;y_{{it}}\geq0)\ \cup\ (x_{{it}}<0,\;y_{{it}}<0){\big)}, \\ &i=1,2,\cdots,n;\;t=1,2,\cdots,T \}, \\ \text{AEz}(a) =& \{(x_{{it}}, y_{{it}})| \; {\big(} (0\leq x_{{it}}\leq a,\;0\leq y_{{it}}\leq a)\\ &\cup\ (-a< x_{{it}}<0,\;-a< y_{{it}}<0){\big)} \\ &i=1,2,\cdots,n;\;t=1,2,\cdots,T \}, \quad \text{and} \\ \text{Ez}(a)=&\{(x_{{it}},y_{{it}})|\;-a\leq x_{{it}},x_{{it}} \leq a,\;-a \\ \leq& y_{{it}},y_{{it}} \leq a,\; t=1,2,\cdots,T\}.\\ \end{aligned} $$

SA is the set of “agreement” pairs of each difference between the values of the gold standard and the experimental technique. Ez(*a*) is the set of pairs plotted in the exclusion zone. In the four-quadrant plot, the exclusion zone (middle square in Fig. 2) is usually placed to remove data plots close to the origin of the plot, because it is difficult to determine whether such small values have occurred because of the examination or random errors (e.g., Critchley et al., [12]). The gray points plotted in the exclusion zone in Fig. 2 are excluded when calculating the concordance rate. The range of the exclusion zone depends on *a*, which is set from a clinical point of view (e.g., Saugel et al., [18]). AEz(*a*) is the set of the “agreement” pairs in the exclusion zone. # signifies the cardinality of a set. The concordance rate in Eq. (1) is the ratio between the number of data points in the “agreement” sections, except the exclusion zone, with all data points that fall outside the exclusion zone.

## Methods

### Proposed concordance rate for the four-quadrant plot

#### General framework of the proposed concordance rate

The proposed concordance rate evaluates the equivalence between the experimental technique and the gold standard through a calculation that considers the individual subjects. This proposed method includes the exclusion zone as well and is defined as the conditional probability. It corresponds to the event falling out of the exclusion zone at all time points. We estimate the parameters of the population with all the data.

The approach for calculation of the proposed method starts with the four-quadrant plot per point *t*. First, the quadrant sections are named *A*_*t*_ to *D*_*t*_. The sample space where the *t*th value falls in each section can be described in four ways:

$$\begin{array}{*{20}l} A_{t}=&\{\omega |\; X_{t} (\omega) \geq 0, Y_{t} (\omega) \geq 0\}, \\ B_{t}=&\{\omega |\; X_{t} (\omega) < 0, Y_{t} (\omega) < 0\}, \\ C_{t}=&\{\omega |\; X_{t} (\omega) < 0, Y_{t} (\omega) \geq 0\}, \quad \text{and} \\ D_{t}=&\{\omega |\; X_{t} (\omega) \geq 0, Y_{t} (\omega) < 0\} \quad (t = 1, 2, \cdots, T). \end{array} $$

Here, *X*_*t*_ and *Y*_*t*_ are random variables of each difference of the values of the gold standard and experimental techniques, respectively. *X*_*t*_ and *Y*_*t*_ correspond to *x*_*it*_ and *y*_*it*_, respectively. X=(*X*_1_,*X*_2_,⋯,*X*_*T*_) and Y=(*Y*_1_,*Y*_2_,⋯,*Y*_*T*_) are assumed to be distributed from multivariate normal distribution. *A*_*t*_ in the upper-right and *B*_*t*_ in the lower-left quadrants of the four-quadrant plot (Fig. 2) correspond with “agreement,” whereas *C*_*t*_ in the upper-left and *D*_*t*_ in the lower-right quadrants are in “disagreement.”

Here, the family of sets is defined as follows:

$$\begin{array}{*{20}l} \mathscr{W}_{t} = \{A_{t} \cup B_{t}, C_{t} \cup D_{t}\} \quad (t = 1, 2, \cdots, T). \end{array} $$

Then, exclusion zone at the *t*th time is

$$\begin{aligned} \mathrm{Ez_{t}}(a)=&\{\omega|\;-a \leq X_{t}(\omega) \leq a, -a \leq Y_{t}(\omega) \leq a\} \quad (t = 1, 2, \cdots, T). \end{aligned} $$

Ez(*a*) is also divided into four-quadrant sections:

$$\begin{aligned} \mathrm{EzA_{t}}(a)=&\{\omega|\;0 \leq X_{t}(\omega) \leq a, 0 \leq Y_{t}(\omega) \leq a\}, \\ \mathrm{EzB_{t}}(a)=&\{\omega|\;-a \leq X_{t}(\omega) \leq 0, -a \leq Y_{t}(\omega) \leq 0\}, \\ \mathrm{EzC_{t}}(a)=&\{\omega|\;-a \leq X_{t}(\omega) \leq 0, 0 \leq Y_{t}(\omega) \leq a\}, \\ \mathrm{EzD_{t}}(a)=&\{\omega|\;0 \leq X_{t}(\omega) \leq a, -a \leq Y_{t}(\omega) \leq 0\}\quad (t = 1, 2, \cdots, T). \end{aligned} $$

The assets of the random variables in *A*_*t*_,*B*_*t*_,*C*_*t*_, and *D*_*t*_, except the exclusion zone, are defined as follows:

$$\begin{array}{*{20}l} A_{t}^{\dagger} =& A_{t} \cap \mathrm{EzA_{t}}(a)^{c}, \\ B_{t}^{\dagger} =& B_{t} \cap \mathrm{EzB_{t}}(a)^{c}, \\ C_{t}^{\dagger} =& C_{t} \cap \mathrm{EzC_{t}}(a)^{c}, \quad \text{and}\\ D_{t}^{\dagger} =& D_{t} \cap \mathrm{EzD_{t}}(a)^{c}, \end{array} $$

where *Z*^*c*^ is the complement of arbitrary set *Z*. $A_{t}^{\dagger }$ and $B_{t}^{\dagger }$ are the events of “agreement” that do not fall into the exclusion zone, whereas $C_{t}^{\dagger }$ and $D_{t}^{\dagger }$ are the events of “disagreement” out of the exclusion zone.

The proposed concordance rate is calculated in the condition in which all pairs of (*X*_*t*_,*Y*_*t*_) are not in the exclusion zone. That is, if any pair of events for that subject drops to the exclusion zone at least once, these events are excluded from the calculation of proposed concordance rate. This can be described as

$$\begin{array}{*{20}l} \text{NEz}(a) = \Big \{ \omega|\; \forall t \; (t = 1,2, \cdots, T); \; \omega \notin \text{Ez}_{t}(a)\Big \}. \end{array} $$

Here, the two clinical testing methods are regarded as equivalent if *X*_*t*_ and *Y*_*t*_ show the same direction of trends more than *m* times out of *T* times per subject. *m* is determined from a clinical perspective. *T* is the number of differences of measurement values. Given this idea, we propose the new concordance rate, in which the probability of “agreement” of more than *m* times in *T* is defined as follows:

2 $$\begin{array}{*{20}l} P\left[ \bigcup_{t = m}^{T} H_{t} | \text{NEz}(a) \right]  \ =& \frac{ P\left[ (\bigcup_{t = m}^{T} H_{t}) \cap \text{NEz}(a) \right] } { P\left [ \text{NEz}(a) \right] }\\ =& \frac{ \sum_{t = m}^{T} P\left[ H_{t} \cap \text{NEz}(a) \right] } { 1 - P\left [ \bigcup_{s=1}^{T} \mathrm{Ez_{s}}(a) \right] },  \end{array} $$

where

3 $$ \begin{aligned} H_{t} &= \left\{ \omega |\; (W_{1}(\omega), W_{2}(\omega), \cdots, W_{T}(\omega)) \in \prod_{s=1}^{T} \mathscr{W}_{s}, \sum_{s=1}^{T} I(W_{s}(\omega) \right.\\&\left.= A_{s}(\omega) \cup B_{s}(\omega)) = t {\vphantom{\prod_{s=1}^{T}}}\right\}.  \end{aligned}  $$

*H*_*t*_ in Eq. (2) is the subset of the sample space in which the trend between *X* and *Y* agrees *t* times. *I* is the indicator function in the condition in which the *s*th data fall in *A*^*†*^ or *B*^*†*^. $\prod _{s=1}^{T} \mathscr {W}_{s}$ in Eq. (3) indicates the product.

#### Example of the proposed index, t = 2

Next, we explain the proposed concordance rate in the case of *m*=1 and *T*=2, that is, at three points in time. The probability can be calculated as follows:

4 $$\begin{array}{*{20}l} P\Big[ \bigcup_{t = 1}^{2} H_{t} | \text{NEz}(a) \Big] \ = \frac{ \sum_{t = 1}^{2} P\Big[ H_{t} \cap \text{NEz}(a) \Big] } { 1 - P\left[ \bigcup_{s=1}^{2} \text{Ez}_{s}(a) \right] }.  \end{array} $$

The reason why we show the example of the proposed concordance rate in Eq. (4) is to show the way of calculating the proposed concordance rate in practical. At first, the proposed concordance rate is calculated based on normal distribution. Therefore, it needs transformation of the description of the proposed concordance rate to calculate the probability by using integral calculus based on the density function. Next, such the calculation becomes a little complicated due to the combination. Through the example of the case *T*=2, we provide how to calculate the proposed concordance rate.

We apply the definition at *T*=2 to a four-quadrant plot. There are three patterns in the case of *T*=2: agreement in *t*=1, agreement in *t*=2, and agreements in *t*=1 and *t*=2. The probability of the numerator in the definition formula is

5 $$ {}\begin{aligned} P[H_{1} \cap \text{NEz}(a)] =& P[(A_{1}^{\dagger} \cup B_{1}^{\dagger}) \cap (C_{2}^{\dagger} \cup D_{2}^{\dagger})] \\&+ P[(C_{1}^{\dagger} \cup D_{1}^{\dagger}) \cap (A_{2}^{\dagger} \cup B_{2}^{\dagger})] \end{aligned}  $$

6 $$ {}\begin{aligned} P[H_{2} \cap \text{NEz}(a)] =& P[(A_{1}^{\dagger} \cup B_{1}^{\dagger}) \cap (A_{2}^{\dagger} \cup B_{2}^{\dagger})].  \end{aligned}  $$

**Fig. 3:**
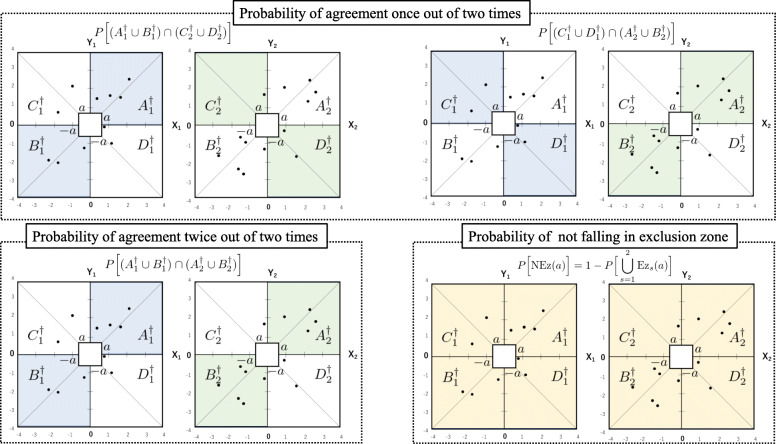
Image of the proposed concordance rate such that at least one agreement out of two times measurement

For the image of the proposed method described in Eq. (5) and Eq. (6), see Fig. 3.

To describe each case, the range in which the data point enters into each quadrant of the plot is set as *F*={[0,*∞*]^*T*^, [−*∞*,0]^*T*^}, and the range of the exclusion zone is *E*={[0,*a*]^*T*^, [−*a*,0]^*T*^}. The vectors to describe the range for the probability calculations are as follows:

$$\begin{aligned} \boldsymbol{v_{1}} = \left[ \begin{array}{c} v_{11} \\ v_{21} \\ \end{array} \right], \quad \boldsymbol{v_{2}} = \left[ \begin{array}{c} v_{12} \\ v_{22} \\ \end{array} \right], \quad \boldsymbol{z_{1}} = \left[ \begin{array}{c} z_{11} \\ z_{21} \\ \end{array} \right], \quad \boldsymbol{z_{2}} = \left[ \begin{array}{c} z_{12} \\ z_{22} \\ \end{array} \right], \end{aligned} $$

where ***v***_1_, ***v***_2_, ***z***_1_, ***z***_2_ are able to take the elements of *F* or *E*. The first term of Eq. (5) is the probability with which the trend of *X*_1_ and *Y*_1_ is in agreement, whereas that of *X*_2_ and *Y*_2_ is not. This can also be expressed as

7 $$ {}\begin{aligned} &P\left[ (A_{1}^{\dagger} \cup B_{1}^{\dagger}) \cap (C_{2}^{\dagger} \cup D_{2}^{\dagger}) \right] \\ =&\sum_{\substack{\boldsymbol{v_{1}} = \boldsymbol{z_{1}}, \boldsymbol{v_{2}} \neq \boldsymbol{z_{2}} \\ \boldsymbol{v_{1}}, \boldsymbol{v_{2}}, \boldsymbol{z_{1}}, \boldsymbol{z_{2}} \in F }} P(v_{11} < X_{1} < v_{21},\; v_{12} < X_{2} < v_{22},\; z_{11} < Y_{1}\\& < z_{21},\; z_{12} < Y_{2} < z_{22}) \\ &+ \sum_{\substack{\boldsymbol{v_{1}} = \boldsymbol{z_{1}}, \boldsymbol{v_{2}} \neq \boldsymbol{z_{2}} \\ \boldsymbol{v_{1}},\boldsymbol{v_{2}}, \boldsymbol{z_{1}}, \boldsymbol{z_{2}} \in E }} P(v_{11} < X_{1} < v_{21},\; v_{12} < X_{2} < v_{22},\; z_{11}\\& < Y_{1} < z_{21},\; z_{12} < Y_{2} < z_{22}) \\ &- \sum_{\substack{\boldsymbol{v_{1}} = \boldsymbol{z_{1}}, \boldsymbol{v_{2}} \neq \boldsymbol{z_{2}} \\ \boldsymbol{v_{1}}, \boldsymbol{z_{1}} \in F, \; \boldsymbol{v_{2}}, \boldsymbol{z_{2}} \in E }} P(v_{11} < X_{1} < v_{21},\; v_{12} < X_{2} < v_{22},\; z_{11}\\& < Y_{1} < z_{21},\; z_{12} < Y_{2} < z_{22}) \\ &-\sum_{\substack{\boldsymbol{v_{1}} = \boldsymbol{z_{1}}, \boldsymbol{v_{2}} \neq \boldsymbol{z_{2}} \\ \boldsymbol{v_{1}}, \boldsymbol{z_{1}} \in E, \; \boldsymbol{v_{2}}, \boldsymbol{z_{2}} \in F }} P(v_{11} < X_{1} < v_{21},\; v_{12} < X_{2} < v_{22},\; z_{11}\\& < Y_{1} < z_{21},\; z_{12} < Y_{2} < z_{22}).  \end{aligned}  $$

Then, the second term of Eq. (5) is the probability when the trend of *X*_1_ and *Y*_1_ is in disagreement, but that of *X*_2_ and *Y*_2_ is in agreement. This can be rewritten similarly as

8 $$ {}\begin{aligned} &P\left[ (C_{1}^{\dagger} \cup D_{1}^{\dagger}) \cap (A_{2}^{\dagger} \cup B_{2}^{\dagger}) \right] \\ =&\sum_{\substack{\bf{v}_{1} \neq \boldsymbol{z_{1}}, \boldsymbol{v_{2}} = \boldsymbol{z_{2}} \\ \boldsymbol{v_{1}}, \boldsymbol{v_{2}}, \boldsymbol{z_{1}}, \boldsymbol{z_{2}} \in F }} P(v_{11} < X_{1} < v_{21},\; v_{12} < X_{2} < v_{22},\; z_{11} < Y_{1}\\& < z_{21},\; z_{12} < Y_{2} < z_{22})\\ &+ \sum_{\substack{\boldsymbol{v_{1}} \neq \boldsymbol{z_{1}}, \boldsymbol{v_{2}} = \boldsymbol{z_{2}} \\ \boldsymbol{v_{1}}, \boldsymbol{v_{2}}, \boldsymbol{z_{1}}, \boldsymbol{z_{2}} \in E }} P(v_{11} < X_{1} < v_{21},\; v_{12} < X_{2} < v_{22},\; z_{11} \\&< Y_{1} < z_{21},\; z_{12} < Y_{2} < z_{22}) \\ &- \sum_{\substack{\boldsymbol{v_{1}} \neq \boldsymbol{z_{1}}, \boldsymbol{v_{2}} = \boldsymbol{z_{2}} \\ \boldsymbol{v_{1}}, \boldsymbol{z_{1}} \in F, \; \boldsymbol{v_{2}}, \boldsymbol{z_{2}} \in E }} P(v_{11} < X_{1} < v_{21},\; v_{12} < X_{2} < v_{22},\; z_{11} \\&< Y_{1} < z_{21},\; z_{12} < Y_{2} < z_{22}) \\ &- \sum_{\substack{\boldsymbol{v_{1}} \neq \boldsymbol{z_{1}}, \boldsymbol{v_{2}} = \boldsymbol{z_{2}} \\ \boldsymbol{v_{1}}, \boldsymbol{z_{1}} \in E, \; \boldsymbol{v_{2}}, \boldsymbol{z_{2}} \in F }} P(v_{11} < X_{1} < v_{21},\; v_{12} < X_{2} < v_{22},\; z_{11} \\&< Y_{1} < z_{21},\; z_{12} < Y_{2} < z_{22}).  \end{aligned}  $$

Equation (6) is the probability that the trends of *X*_1_ and *Y*_1_ and of *X*_2_ and *Y*_2_ are both concordant:

9 $$ {}\begin{aligned} &P\left[ (A_{1}^{\dagger} \cup B_{1}^{\dagger}) \cap (A_{2}^{\dagger} \cup B_{2}^{\dagger}) \right] \\ =&\sum_{\substack{\boldsymbol{v_{1}} = \boldsymbol{z_{1}}, \boldsymbol{v_{2}} = \boldsymbol{z_{2}} \\ \boldsymbol{v_{1}}, \boldsymbol{v_{2}}, \boldsymbol{z_{1}}, \boldsymbol{z_{2}} \in F }} P(v_{11} < X_{1} < v_{21},\; v_{12} < X_{2} < v_{22},\; z_{11} < Y_{1}\\& < z_{21},\; z_{12} < Y_{2} < z_{22}) \\ &+ \sum_{\substack{\boldsymbol{v_{1}} = \boldsymbol{z_{1}}, \boldsymbol{v_{2}} = \boldsymbol{z_{2}} \\ \boldsymbol{v_{1}}, \boldsymbol{v_{2}}, \boldsymbol{z_{1}}, \boldsymbol{z_{2}} \in E }} P(v_{11} < X_{1} < v_{21},\; v_{12} < X_{2} < v_{22},\; z_{11} \\&< Y_{1} < z_{21},\; z_{12} < Y_{2} < z_{22}) \\ &- \sum_{\substack{\boldsymbol{v_{1}} = \boldsymbol{z_{1}}, \boldsymbol{v_{2}} = \boldsymbol{z_{2}} \\ \boldsymbol{v_{1}}, \boldsymbol{z_{1}} \in F, \; \boldsymbol{v_{2}}, \boldsymbol{z_{2}} \in E }} P(v_{11} < X_{1} < v_{21},\; v_{12} < X_{2} < v_{22},\; z_{11}\\& < Y_{1} < z_{21},\; z_{12} < Y_{2} < z_{22}) \\ &- \sum_{\substack{\boldsymbol{v_{1}} = \boldsymbol{z_{1}}, \boldsymbol{v_{2}} = \boldsymbol{z_{2}} \\ \boldsymbol{v_{1}}, \boldsymbol{z_{1}} \in E, \; \boldsymbol{v_{2}}, \boldsymbol{z_{2}} \in F }} P(v_{11} < X_{1} < v_{21},\; v_{12} < X_{2} < v_{22},\; z_{11}\\& < Y_{1} < z_{21},\; z_{12} < Y_{2} < z_{22}).  \end{aligned}  $$

Finally, the probability of the denominator in *T*=2 is

10 $$ \begin{aligned} 1 &- P\left[ \bigcup_{s=1}^{2} \text{Ez}_{s}(a) \right] \\ =1&- P(-a < X_{1} \!< a, -\infty < X_{2} \!<\infty,-a < Y_{1} \!< a, -\infty < Y_{2} <\infty) \\ &- P(-\infty < X_{1} \!< \infty, -a < X_{2} \!< a,-\infty < Y_{1} \!< \infty, -a < Y_{2} < a)\\ &+ P(-a < X_{1} < a, -a < X_{2} < a,\;-a < Y_{1} < a, -a < Y_{2} < a).  \end{aligned}  $$

In the proposed concordance rate, we assume that all random variables are distributed from multivariate normal distribution. Therefore, we must estimate the mean vectors and covariance matrices to calculate the concordance rate. The method of estimating these parameters is described in the next subsection.

#### Estimation of the proposed concordance rate

First, we define *Z*=(*X*_1_, ⋯, *X*_*T*_, *Y*_1_, ⋯, *Y*_*T*_)=(*Z*_1_, ⋯, *Z*_*T*_,*Z*_*T*+1_, ⋯, *Z*_*T*+*T*_). Since the proposed method assumes that *Z* are distributed from *T*+*T*-dimensional normal distribution, it is necessary to estimate the *T*+*T*-dimensional mean vector and variance covariance matrix to calculate the concordance rate. The estimated mean vector in the proposed approach is $\bar {\boldsymbol {z}}=(\bar {x}_{1},\;\cdots,\;\bar {x}_{T},\; \bar {y}_{1},\;\cdots,\;\bar {y}_{T})^{T}$, where $\bar {x}_{t}$ and $\bar {y}_{t}$ are the mean of the *t*th value of gold standard and experimental technique, respectively. The covariance matrix based on the differences between the times is $\phantom {\dot {i}\!}{\boldsymbol {S}} = (s_{tt^{\dagger }})\quad (t,t^{\dagger }=1,2,\cdots,T+T)$, where $\phantom {\dot {i}\!}s_{tt^{\dagger }}$ is the covariance between *t* and *t*^*†*^. By using these estimators, the proposed concordance rate in Eq (2), defined as the conditional probability $P[ \bigcup _{t = m}^{T} H_{t} | \text {NEz}(a)]$, can be calculated. When calculating the mean vector and covariance matrix, data in the exclusion zone are also used, while the effect of the exclusion zone is considered under the conditional probability. The estimation of the mean vector and the covariance matrix in the proposed concordance rate is expected to be stable, therefore small sample sizes may have less impact than that of the conventional concordance rate.

Next, we show the practical procedure of calculating the proposed concordance rate with *T*=2 and *m*=2 as an example.

Estimation of the proposed concordance rate with *T*=2 and *m*=2 Step1: Set *a*. Step2: From data of a gold standard $\boldsymbol {x}_{i}^{*} = (x_{i1}^{*},\; x_{i2}^{*},\; x_{i3}^{*})^{T} \; (i=1,2,\cdots,n)$ and of a experimental technique $\boldsymbol {y}_{i}^{*} = (y_{i1}^{*},\; y_{i2}^{*},\; y_{i3}^{*})^{T} \; (i=1,2,\cdots,n)$, each difference vector is obtained as follows:

$$\begin{aligned} &\boldsymbol{x}_{i} = (x_{i1}, x_{i2})^{T} = (x_{i2}^{*}, x_{i3}^{*})^{T} - (x_{i1}^{*}, x_{i2}^{*})^{T} \\ \text{and} \quad &\boldsymbol{y}_{i} = (y_{i1},\; y_{i2})^{T} = (y_{i2}^{*},\; y_{i3}^{*})^{T} - (y_{i1}^{*},\; y_{i2}^{*})^{T}, \quad \text{respectively.} \end{aligned} $$

Step3: Let $\boldsymbol {z}_{i} = (\boldsymbol {x}_{i}^{T},\; \boldsymbol {y}_{i}^{T})^{T} \; (i=1,2,\cdots,n)$ and calculate mean vectors and covariance matrix as follows;

$$\begin{array}{*{20}l}  \boldsymbol{z} = \frac{1}{n} \sum_{i=1}^{n} \boldsymbol{z}_{i} \quad \text{and} \quad \boldsymbol{S} = \frac{1}{n-1} \sum_{i=1}^{n} (\boldsymbol{z}_{i} - \bar{\boldsymbol{z}})(\boldsymbol{z}_{i} - \bar{\boldsymbol{z}})^{T}. \end{array} $$

Step4: Calculate the proposed concordance rate $P[ \bigcup _{t = 2}^{2} H_{t} | \text {NEz}(a)] = P[ H_{2} | \text {NEz}(a)]$. First, Eq. (9) is calculated as follows;

$$ \begin{aligned} &P\left[ (A_{1}^{\dagger} \cup B_{1}^{\dagger}) \cap (A_{2}^{\dagger} \cup B_{2}^{\dagger}) \right]  \\ =&\sum_{\substack{\boldsymbol{v_{1}} = \boldsymbol{z_{1}}, \boldsymbol{v_{2}} = \boldsymbol{z_{2}} \\ \boldsymbol{v_{1}}, \boldsymbol{v_{2}}, \boldsymbol{z_{1}}, \boldsymbol{z_{2}} \in F }} \int_{v_{11}}^{v_{21}} \int_{v_{12}}^{v_{22}} \int_{z_{11}}^{z_{21}} \int_{z_{12}}^{z_{22}} f(\boldsymbol{z};\bar{\boldsymbol{z}}, \boldsymbol{S}) d\boldsymbol{z}  \\ &+ \sum_{\substack{\boldsymbol{v_{1}} = \boldsymbol{z_{1}}, \boldsymbol{v_{2}} = \boldsymbol{z_{2}} \\ \boldsymbol{v_{1}}, \boldsymbol{v_{2}}, \boldsymbol{z_{1}}, \boldsymbol{z_{2}} \in E }} \int_{v_{11}}^{v_{21}} \int_{v_{12}}^{v_{22}} \int_{z_{11}}^{z_{21}} \int_{z_{12}}^{z_{22}} f(\boldsymbol{z};\bar{\boldsymbol{z}}, \boldsymbol{S}) d\boldsymbol{z}  \\ &- \sum_{\substack{\boldsymbol{v_{1}} = \boldsymbol{z_{1}}, \boldsymbol{v_{2}} = \boldsymbol{z_{2}} \\ \boldsymbol{v_{1}}, \boldsymbol{z_{1}} \in F, \; \boldsymbol{v_{2}}, \boldsymbol{z_{2}} \in E }} \int_{v_{11}}^{v_{21}} \int_{v_{12}}^{v_{22}} \int_{z_{11}}^{z_{21}} \int_{z_{12}}^{z_{22}} f(\boldsymbol{z};\bar{\boldsymbol{z}}, \boldsymbol{S}) d\boldsymbol{z}  \\ &- \sum_{\substack{\boldsymbol{v_{1}} = \boldsymbol{z_{1}}, \boldsymbol{v_{2}} = \boldsymbol{z_{2}} \\ \boldsymbol{v_{1}}, \boldsymbol{z_{1}} \in E, \; \boldsymbol{v_{2}}, \boldsymbol{z_{2}} \in F }} \int_{v_{11}}^{v_{21}} \int_{v_{12}}^{v_{22}} \int_{z_{11}}^{z_{21}} \int_{z_{12}}^{z_{22}} f(\boldsymbol{z};\bar{\boldsymbol{z}}, \boldsymbol{S}) d\boldsymbol{z}  \end{aligned}  $$

where $f(\boldsymbol {z}; \bar {\boldsymbol {z}}, \boldsymbol {S})$ is described as density function of four dimensional normal distribution with $\bar {\boldsymbol {z}}$ and ***S***. For example, each probability in Eq. (11) can be calculated by using the function pmvnorm with the package mvtnorm of statistical software R. Next, as the same manner of Eq. (11), Eq. (10) is calculated as follows;

11 $$ \begin{aligned} &1 - P\left[ \bigcup_{s=1}^{2} \text{Ez}_{s}(a) \right] \\ =&1- \int_{-a}^{a} \int_{-\infty}^{\infty} \int_{-a}^{a} \int_{-\infty}^{\infty} f(\boldsymbol{z};\bar{\boldsymbol{z}}, \boldsymbol{S}) d\boldsymbol{z} - \int_{-\infty}^{\infty} \int_{-a}^{a} \int_{-\infty}^{\infty} \int_{-a}^{a} f(\boldsymbol{z};\bar{\boldsymbol{z}}, \boldsymbol{S}) d\boldsymbol{z} \\ &+ \int_{-a}^{a} \int_{-a}^{a} \int_{-a}^{a} \int_{-a}^{a} f(\boldsymbol{z};\bar{\boldsymbol{z}}, \boldsymbol{S}) d\boldsymbol{z}.  \end{aligned}  $$

Finally by using Eq. (11) and Eq. (11), the proposed concordance is calculated as follows;

$$\begin{array}{*{20}l} P\Big[ H_{2} | \text{NEz}(a) \Big] \ = \frac{ P\left[ H_{2} \cap \text{NEz}(a) \right] } { 1 - P\left[ \bigcup_{s=1}^{2} \text{Ez}_{s}(a) \right] }. \end{array} $$

In this example, we show the case of *m*=2. The case of *m*=1 also can be calculated in the same manner. In the case of *m*=1, it needs to calculate the probability of Eq. (7) and Eq. (8) as the same way of Eq. (11).

### Numerical simulation design

In this subsection, we describe the simulation design including several factor setting (Table 1). We generate the artificial data with the true trend, and compare the diagnosability between the proposed method and the control methods. The detail of the control methods will be explained in Factor 7 below. The true trend is defined as the labels in Table 2 determined by pair of population means of each difference between two consecutive measurements values. The evaluation in this numerical simulation consists of two steps. First, we calculate the ROC curves, and use Are Under the Curve (AUC) (e.g., Pepe, [15]) as the assessment of the diagnosability. In the second step, the cutoff values of each method are computed by Youden’s index (Youden, [20]), and the estimated concordance rates are evaluated based on the cutoff values by factor mentioned below. In this simulation, we used RStudio Version 1.1.453.

**Table 1:** Factors of the simulation design

Factor No.	Factor name	Levels
Factor 1	Means	30
Factor 2	Covariance between the difference values within each measurement method	3
Factor 3	Covariance between *X* and *Y*	2
Factor 4	Number of agreements	2
Factor 5	Exclusion zone	2
Factor 6	Number of subjects	2
Factor 7	Methods	4

**Table 2:** Mean patterns in factor 1: label1 ∘ indicates the pattern of agreement between *μ*_*X*_ and *μ*_*Y*_ two times, otherwise ×. ∘ of label2 means the pattern of agreement more than once in twice, otherwise ×

Pattern No.	*μ*_*X*1_	*μ*_*X*2_	*μ*_*Y*1_	*μ*_*Y*2_	Label1	Label2
Pattern1	-1.5	-1.5	1.5	1.5	×	×
Pattern2	-0.5	-0.5	0.5	0.5	×	×
Pattern3	-1.5	1.5	1.5	1.5	×	∘
Pattern4	0.5	-0.5	0.5	0.5	×	∘
Pattern5	1.5	1.5	1.5	1.5	∘	∘
Pattern6	0.5	0.5	0.5	0.5	∘	∘
Pattern7	-0.5	-1.5	0.5	1.5	×	×
Pattern8	0.5	-1.5	0.5	1.5	×	∘
Pattern9	-0.5	1.5	0.5	1.5	×	∘
Pattern10	0.5	1.5	0.5	1.5	∘	∘
Pattern11	-1.5	-1.5	-1.5	-1.5	∘	∘
Pattern12	-0.5	-0.5	-0.5	-0.5	∘	∘
Pattern13	-1.5	1.5	-1.5	-1.5	×	∘
Pattern14	0.5	-0.5	-0.5	-0.5	×	∘
Pattern15	1.5	1.5	-1.5	-1.5	×	×
Pattern16	0.5	0.5	-0.5	-0.5	×	×
Pattern17	-0.5	-1.5	-0.5	-1.5	∘	∘
Pattern18	0.5	-1.5	-0.5	-1.5	×	∘
Pattern19	-0.5	1.5	-0.5	-1.5	×	∘
Pattern20	0.5	1.5	-0.5	-1.5	×	×
Pattern21	-1.5	-1.5	-1.5	1.5	×	∘
Pattern22	-0.5	-0.5	-0.5	0.5	×	∘
Pattern23	-1.5	1.5	-1.5	1.5	∘	∘
Pattern24	0.5	-0.5	-0.5	0.5	×	×
Pattern25	1.5	1.5	-1.5	1.5	×	∘
Pattern26	0.5	0.5	-0.5	0.5	×	∘
Pattern27	-0.5	-1.5	-0.5	1.5	×	∘
Pattern28	0.5	-1.5	-0.5	1.5	×	×
Pattern29	-0.5	1.5	-0.5	1.5	∘	∘
Pattern30	0.5	1.5	-0.5	1.5	×	∘

We set *T*=2, and the data generation procedure is as follows:

$$\begin{array}{*{20}l} \bf{Z} \sim N (\bf{\mu}_{z}, \Sigma_{z}) \end{array} $$

where ***Z***=(*X*_1_,*X*_2_,*Y*_1_,*Y*_2_)^*T*^. *X*_*t*_ is the difference in the measurement values of the gold standard between the *t*th and (*t*+1)th times (*t*=1,2), and *Y*_*t*_ is that of the experimental technique.

In addition,

$$\begin{array}{*{20}l} \boldsymbol{\mu}_{Z} = \left[ \begin{array}{c} \boldsymbol{\mu}_{X}\\ \boldsymbol{\mu}_{Y}\\ \end{array} \right], \quad \boldsymbol{\Sigma}_{Z} = \left[ \begin{array}{cc} \boldsymbol{\Sigma}_{X} & \boldsymbol{\Sigma}_{{XY}} \\ \boldsymbol{\Sigma}_{{XY}} & \boldsymbol{\Sigma}_{Y}\\ \end{array} \right], \end{array} $$

where *μ*_*X*_=(*μ*_*x*1_,*μ*_*x*2_)^*T*^ and *μ*_*Y*_=(*μ*_*y*1_,*μ*_*y*2_)^*T*^ are the mean vectors of the gold standard and experimental technique, and *Σ*_*X*_ and *Σ*_*Y*_ are the covariance matrices, respectively.

Here,

$$\begin{aligned} \boldsymbol{\Sigma}_{X} = \left[ \begin{array}{cc} {\sigma}_{x1} & {\rho} \\ {\rho} & {\sigma}_{x2}\\ \end{array} \right],\ \ \boldsymbol{\Sigma}_{Y} = \left[ \begin{array}{cc} {\sigma}_{y1} & {\rho} \\ {\rho} & {\sigma}_{y2}\\ \end{array} \right], \ \text{and} \ \quad \boldsymbol{\Sigma}_{{XY}} = \left[ \begin{array}{cc} {\rho}_{{XY}} & {\rho}_{{XY}} \\ {\rho}_{{XY}} & {\rho}_{{XY}}\\ \end{array} \right]. \end{aligned} $$

We set *σ*_*x*1_=*σ*_*x*2_=*σ*_*y*1_=*σ*_*y*2_=1.

Factors set in the simulation are presented in Table 1. The number of patterns for *m*=1 is 30 (Factor1)×3 (Factor2)×2 (Factor3)×1 (Factor4)×2 (Factor5)×2 (Factor6)×3 (Factor7)=2160, and that for *m*=2 is 30 (Factor1)×3 (Factor2)×2 (Factor3)×1 (Factor4)×2 (Factor5)×2 (Factor6)×4 (Factor7)=2880. Thus, the total number of patterns is 2160+2880=5040. For each pattern, corresponding artificial data are generated 100 times, and we evaluate the results. The levels of the seven factors are set as follows.

**Factor 1: Means**

The mean is of 30 patterns, as shown in Table 2. The setting depends on the combination of the magnitude of the mean value and the direction of change in *x* and *y*.

**Factor 2: Covariance between the difference values within each measurement method**

The covariance within each measurement method of the difference values, *ρ*, is set as 0, 1/3, and 2/3 in both *X* and *Y*.

**Factor 3: Covariance between** ***X*** **and** ***Y***

*ρ*_*XY*_=0 and 1/3.

**Factor 4: Number of agreements**

Factor 4 is the number of trending agreements between *X* and *Y*. We set two different situations as follows: (1) agreement more than once in *T*=2, and (2) agreement at both time points.

**Factor 5: Exclusion zone**

*a* of the exclusion zone Ez(*a*) is set as 0.5 and 1.0.

**Factor 6: Number of subjects**

The number of subjects is set as 15 and 40.

**Factor 7: Methods**

We calculate the concordance rate using four methods. CCR, control1, control2, and the proposed method are used in *m*=2, and control1, control2, and the proposed method are used in *m*=1. We denote the proposed concordance rate as “proposal.”

Both control1 and control2 are set by ourselves. The aim is to calculate the probability of the agreement more than *m* times out of *T*. The conventional concordance rate can not be simply compared with the proposed method in the case of *m*≠*T*, because it does not consider the repeated measurements. When conditional probability based on the binomial distribution, which is the formula of the conventional concordance rate, extends to the probability of the agreement more than *m* times out of *T*, we can obtain control1 and control2 as the natural extension.

Control1, based on binomial distribution, is calculated as follows:

$$\begin{array}{*{20}l} \sum_{s=m}^{2} {~\!~\!}_{2}C_{s} p^{s}(1-p)^{(2-s)}, \end{array} $$

where _2_*C*_*s*_ indicates binomial coefficient and

$$\begin{array}{*{20}l} p = \frac{k_{1}+k_{2}}{n_{1}^{\dagger}+n_{2}^{\dagger}}. \end{array} $$

*k*_*t*_ (*t*=1,2) is the number of data that show the same trend between *X*_*t*_ and *Y*_*t*_ out of the exclusion zone. $n_{t}^{\dagger }$ is the number of subjects whose data points fall out of the exclusion zone. The concordance rate in control2 is calculated by the probability at each agreement: twice in two time points is *p*_1_*p*_2_, and once in two time points *p*_1_(1−*p*_2_)+(1−*p*_1_)*p*_2_, where

$$\begin{array}{*{20}l} p_{t} = \frac{k_{t}}{n_{t}^{\dagger}}\quad (t=1,2). \end{array} $$

Subjects whose difference value falls in the exclusion zone of the four-quadrant plot even once are excluded from the calculation of the concordance rate in both control1 and control2 in the same manner as the proposed method.

Next, we explain how to evaluate these results and how to compare them. There are two evaluation indices in this simulation. For the first evaluation index, we label each pattern of means in Table 2. Label1 is the case of *m*=2, and Label2 as *m*=1. In Label1, if *μ*_*X*_ and *μ*_*Y*_ are concordant two times out of two, we mark the corresponding mean pattern as “ ∘”, and the rest as “ ×”. In Label2, the corresponding mean pattern as “ ∘”, if *μ*_*X*_ and *μ*_*Y*_ shows same trend more than once out of two times, otherwise labeled as “ ×”. Then, 1440 (30 (Factor1)×3 (Factor2)×2 (Factor3)×2 (Factor4)×2 (Factor5)×2 (Factor6)) ×100 (the number of iterations) =144000 data in total have these two labels. That means, in the case of *m*=2, 48 ×100 data in each pattern in Table 2 have the same trend label as “ ∘” or “ ×” in Label1 of each pattern. Similarly, for *m*=1, the same data will be given the same label as Label 2. For 144000 data, the concordance rates are calculated by the proposed method, CCR, control1, and control2. With the results of the concordance rates and the labels given to the data, we calculate ROC and AUC (e.g., Pepe, [15]) for each *m*, and compare the AUC values among the proposed method, CCR, control1, and control2.

The second evaluation is the diagnostic performance of the proposed methods and the control methods for each factor. For the factors except Factor 1, the results of the concordance rate methods are compared in each level by the AUC. 144000 data with Label1 and Label2 are split by the levels in each Factor. Then, the AUC of the four concordance rate methods are calculated in *m*=2 and *m*=1. As for Factor 1, data is classified by pattern, which means each level has only one label per *m*. The AUC of Factor 1 can not be calculated, therefore we apply the evaluation below to Factor 1. As the first step, the cutoff value *c*_*mo*_(*m*=1,2;*o*=1,2,3,4) of the concordance rate methods are calculated from ROC by Youden’s index, where each *o* indicates the type of concordance method; the proposed method, CCR, control1 and control2. ROC for each *m* is same as the one in the first evaluation, which computed by the estimated concordance rates and the labels. For example, if the true trend is “ ∘”, the case in that the estimated concordance rate is higher than cutoff value *c*_*mo*_ can be recognized as the proper diagnostic performance. Conversely, if the true trend is “ ∘” and the estimated concordance rate is lower than the cutoff value *c*_*mo*_, the diagnosis is considered incorrect. The case of the label “ ×” is opposite to “ ∘”; the case in that the estimated concordance rate is lower than cutoff value *c*_*mo*_ can be appropriate if the true trend is “ ×”. Specifically, let $\phantom {\dot {i}\!}p^{\dagger }_{i^{*}o}, (i^{*}=1,2,\cdots, n^{*})$ estimated concordance rate, where *n*^∗^ is the number of artificial data aggregated by factor. Here, we set $\phantom {\dot {i}\!}g_{i^{*}}$ such that $\phantom {\dot {i}\!}g_{i^{*}}=1$ if the true trend of *i*^∗^ is “ ∘”, and $\phantom {\dot {i}\!}g_{i^{*}}=0$ if the true trend of *i*^∗^ is “ ×”.

12 $$ \begin{aligned} q_{o} = \frac{\sum_{{i^{*}}=1}^{n^{*}}(g_{{i^{*}}}I(p^{\dagger}_{i^{*}o}\geq c_{{mo}})+(1-g_{{i^{*}}})I(p^{\dagger}_{i^{*}o}< c_{{mo}}))}{n^{*}},\ (o=1,2,3,4) \end{aligned}  $$

where *I* is Indicator function. The Eq. (12) for each method is calculated in *m*=2 and *m*=1, and we compare these results in Factor 1. The value of Eq. (12) closer to 1 is regarded that estimated concordance rate has been evaluated close to the true number of agreement, while the value closer to 0 means that it has not been evaluated correctly.

### Application to sbp data

In this subsection, we show the usefulness of the proposed concordance rate by diagnosability through a real example. The AUC and the ROC curves of the proposed method, CCR, control1, and control2 were compared to evaluate diagnosability.

**Fig. 4:**
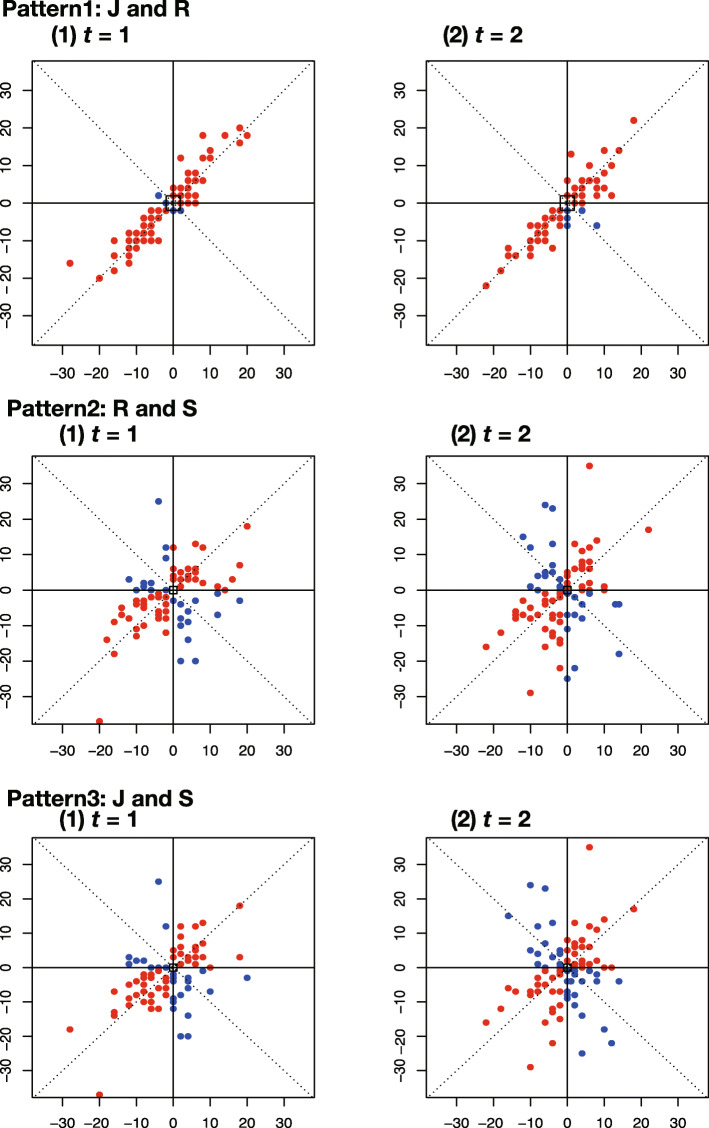
Four-quadrant plots with real example data. Pattern 1: J(observer1) and R(observer2), Pattern 2: R and S(automatic machine), and Pattern 3: J and S

We applied the proposed concordance rate method and the comparative methods to the blood pressure data of package MethComp in R software (Carstensen et al., [8]). The data (Altman and Bland, [2]; Bland and Altman, [5]) comprise the blood pressure measurement for 85 subjects based on 3 types of data: data named as J and R were measured by a gold standard conducted by 2 different human observers, and S was measured by an automatic machine as the experimental method. The study was performed at three time points for each subject. The four-quadrant plots generated from the real data are presented in Fig. 4. Comparing 2 of the 3 measurement results to one another, we find that there are three pairs, namely, J(observer1) and R(observer2), R and S(auto machine), and J and S. Each pattern has two plots, (1) *t*=1 and (2) *t*=2. We calculated the concordance rate with the proposed method, CCR, control1, and control2 for each pair.

For the assessment of the methods, we compared the diagnostic feasibility of the proposed and the conventional methods of CCR, control1, and control2. Specifically, 10 subjects out of 85 were randomly selected as sampling with replacement for calculation with the proposed method, CCR, control1, and control2 in all three patterns. The procedure was iterated 1000 times and the diagnostic performances of each method was evaluated. We chose the parameter *m*=2 in this example, because, in *m*=1, the proposed concordance rate cannot be directly compared with CCR which does not deal with the repeated measurements. As for Ez(*a*), *a* was set as the 10% quantile point of the absolute values for both the gold standard and experimental method (e.g., Critchley et al., [12]).

Each pattern of the four-quadrant plots in Fig. 4 shows the characteristics of the real example. The data of J and R in Pattern 1 have many red points that show “agreement” of the trend between two data points, and most of these points lie close to the 45^∘^ line, because this tendency naturally derives from the same established measurement method. On the other hand, data of S, the experimental measurement, is collected differently, thus the plots of Pattern 2 and Pattern 3 have more blue dots as “disagreement” than the plots of Pattern 1, and the data are distributed with variation. Then, Pattern 1 is set as the “agreement” label, and both Patterns 2 and 3 are as the “disagreement” label. The “agreement” label is given as a true label to the concordance rates of the proposed method, CCR, control1 and control2, calculated with 10 sampling data of Pattern 1, J and R. Similarly, “disagreement” is assigned to each estimated concordance rate using 10 sampling data of Pattern 2 (R and S) and Pattern 3 (J and R), respectively. Here 1000 concordance rates have “agreement” and 2000 have “disagreement” per method. Using these label and the estimated concordance rates of the proposed method, CCR, control1 and control2, we compare ROC and assess AUC which method has the high rate of diagnosability.

## Results

### Simulation results

#### Diagnosability of the estimation of each concordance method

**Fig. 5:**
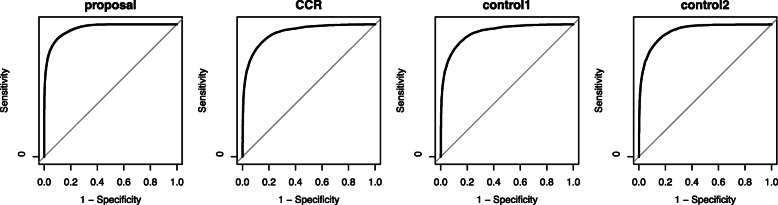
ROC curves of the proposed method, CCR, control1, and control2 for the simulation in the case of *m*=2

**Fig. 6:**
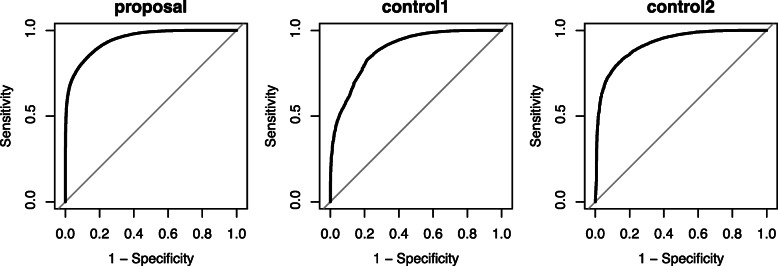
ROC curves of the proposed method, control1, and control2 for the simulation in the case of *m*=1

We described the ROC curves in Figs. 5 and 6, and calculated AUC in Table 3. Figure 5 is the ROC of the proposed method, CCR, control1 and control2 in *m*=2, and the ROC of the proposed method, control1 and control2 in *m*=1 draw in Fig. 6. According to Table 3, the AUC of the proposed method was highest among all compared method including CCR in *m*=2. It indicates that the diagnostic capability of the proposed method was superior to the conventional methods in *m*=2. In *m*=2, the AUC of CCR and control1 was the same, since control1 is an extension of CCR in *m* out of *T*, which is a natural result. As for the case of *m*=1, the AUC of the proposed method was higher than all control methods, control1 and control2. The proposed method in *m*=1 showed the higher diagnostic capability than the conventional methods.

**Table 3:** AUC of the proposed method, CCR, control1, and control2 in the simulation

		*m*=2				*m*=1	
	Proposal	CCR	Control1	Control2	Proposal	Control1	Control2
AUC	0.967	0.938	0.938	0.947	0.945	0.888	0.920

#### Diagnosability of the estimation of each concordance method by factor

Here, we indicate the diagnosability by factor. *q*_*o*_ in Eq. (12) computed by the pattern of Factor 1 is compared between the proposed method and the control concordance rate methods. In *m*=2 of Factor 1, *q*_*o*_ of the compared concordance rate methods are calculated for 48×100 data in each pattern in Table 2. *q*_*o*_ in *m*=1 is also obtained for the proposed method, control1 and control2 from the same number of data. The results of Factor 1 Means is described in Table 4. All the proposed method outperformed than CCR in *m*=2. In the pattern 6,12, and 29, the proposed method was almost same as control2. In case of *m*=1, many of the proposed method had better results than control1 and control2, while control1 was better than the proposed method in the pattern 4,6,10,12,14,22, and 26. The absolute values of true mean in all these patterns includes small value, 0.5.

**Table 4:** The result of the simulation for factor 1: means

		*m*=2				*m*=1	
Pattern No.	Proposal	CCR	Control1	Control2	Proposal	Control1	Control2
Pattern1	1.000	1.000	1.000	1.000	1.000	1.000	1.000
Pattern2	0.929	0.920	0.920	0.879	0.693	0.475	0.671
Pattern3	1.000	0.995	0.995	1.000	0.997	0.884	0.967
Pattern4	0.812	0.766	0.766	0.701	0.714	0.812	0.640
Pattern5	1.000	1.000	1.000	1.000	1.000	1.000	1.000
Pattern6	0.652	0.551	0.551	0.656	0.880	0.947	0.861
Pattern7	1.000	1.000	1.000	0.999	0.983	0.960	0.969
Pattern8	1.000	0.997	0.997	0.996	0.304	0.213	0.253
Pattern9	0.588	0.374	0.374	0.398	0.998	0.992	0.988
Pattern10	0.951	0.933	0.933	0.942	0.998	1.000	0.997
Pattern11	1.000	1.000	1.000	1.000	1.000	1.000	1.000
Pattern12	0.654	0.540	0.540	0.659	0.883	0.951	0.861
Pattern13	1.000	0.997	0.997	0.999	0.998	0.893	0.975
Pattern14	0.810	0.780	0.780	0.695	0.703	0.806	0.648
Pattern15	1.000	1.000	1.000	1.000	1.000	1.000	1.000
Pattern16	0.931	0.922	0.922	0.873	0.700	0.454	0.677
Pattern17	0.958	0.938	0.938	0.945	0.999	0.999	0.997
Pattern18	0.605	0.385	0.385	0.413	0.998	0.986	0.986
Pattern19	1.000	0.996	0.996	0.996	0.302	0.223	0.252
Pattern20	1.000	0.999	0.999	0.999	0.986	0.967	0.969
Pattern21	1.000	0.993	0.993	0.998	0.999	0.895	0.970
Pattern22	0.791	0.765	0.765	0.697	0.683	0.794	0.616
Pattern23	1.000	1.000	1.000	1.000	1.000	1.000	1.000
Pattern24	0.941	0.901	0.901	0.855	0.654	0.432	0.640
Pattern25	1.000	0.992	0.992	0.997	0.997	0.885	0.974
Pattern26	0.801	0.772	0.772	0.705	0.714	0.812	0.635
Pattern27	1.000	0.995	0.995	0.995	0.301	0.216	0.246
Pattern28	1.000	0.999	0.999	0.999	0.985	0.968	0.977
Pattern29	0.910	0.905	0.905	0.920	1.000	0.999	0.997
Pattern30	0.594	0.372	0.372	0.392	0.999	0.990	0.990

Next, in Factor 2, the AUC is calculated for 240×100 in each level per *m* (Table 5). The proposed method was better than the control methods. In *m*=2, the values of the control methods were not changed, and the values of the proposed method have been increased as covariance rises. It showed that the diagnostic performance of the proposed method improved with the rise of covariance, while that of all control methods did not change. In *m*=1, the diagnostic performance of the proposed method increased as covariance risen, while that of the control1 and control2 decreased. The AUC of Factor 3, 5 and 6 were calculated by level as the same manner of Factor 2. The results of Factor 3, 5 and 6 are shown in Tables 6, 7 and 8, respectively. The proposed methods showed higher values of diagnosability than the control methods in any *m* in these factors.

**Table 5:** AUC of the simulation for factor 2: covariance of the difference values within each measurement method

		*m*=2				*m*=1	
	Proposal	CCR	Control1	Control2	Proposal	Control1	Control2
*ρ*=0	0.965	0.937	0.937	0.946	0.941	0.895	0.924
*ρ*=1/3	0.968	0.939	0.939	0.949	0.947	0.889	0.921
*ρ*=2/3	0.972	0.937	0.937	0.946	0.948	0.880	0.916

**Table 6:** AUC of the simulation for factor 3: covariance between *X* and *Y*

		*m*=2				*m*=1	
	Proposal	CCR	Control1	Control2	Proposal	Control1	Control2
*ρ*_*XY*_=0	0.965	0.937	0.937	0.946	0.941	0.895	0.924
*ρ*_*XY*_=1/3	0.968	0.939	0.939	0.949	0.947	0.889	0.921

**Table 7:** AUC of the simulation for factor 5: exclusion zone

		*m*=2				*m*=1	
	Proposal	CCR	Control1	Control2	Proposal	Control1	Control2
*a*=0.5	0.965	0.937	0.937	0.946	0.941	0.895	0.924
*a*=1.0	0.968	0.939	0.939	0.949	0.947	0.889	0.921

**Table 8:** AUC of the simulation for factor 6: number of subjects

		*m*=2			*m*=1		
	Proposal	CCR	Control1	Control2	Proposal	Control1	Control2
*n*=15	0.965	0.937	0.937	0.946	0.941	0.895	0.924
*n*=40	0.968	0.939	0.939	0.949	0.947	0.889	0.921

### Results of sbp data

**Fig. 7:**
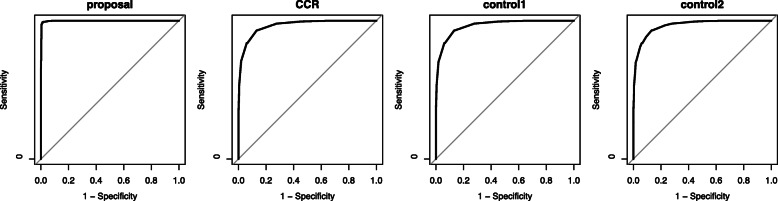
ROC of the proposed method, CCR, control1, and control2 in a real example

The AUC of the proposed method, CCR, control1 and control2 is shown in Table 9. Each concordance rate was estimated with high accuracy in *m*=2 of the example data, meanwhile the proposed method was better than the comparative concordance rate methods. As for the ROC curves in Fig. 7, the plot of the proposed method drew a curve with an almost-right angle, while the curve was more moderate in the ROC of CCR. These curves indicate that the proposed approach has more accuracy than the conventional concordance rates.

**Table 9:** AUC of the proposed method, CCR, control1, and control2 in a real example

Proposal	CCR	Control1	Control2
0.999	0.964	0.964	0.965

## Discussion

The conventional concordance rate for a four-quadrant plot is one of the methods for evaluating the equivalence between a new testing method and standard measurement method. In many clinical practices, these values are observed repeatedly for the same subjects. However, the conventional concordance rate for the four-quadrant plot does not consider when evaluating the trend of measurement values between the two clinical testing methods being compared. Therefore, we proposed a new concordance rate based on normal distribution that is calculated using the difference in values of each measurement technique depending on the choice of *m* hyper parameter as the minimum number of agreements to evaluate the equivalence.

The diagnosability of the estimation of the proposed method was superior to those of CCR, control1, and control2 according to the results of the numerical simulations. The results for each factor were also better for the proposed method than for the control methods. In Factor 2 covariance within the individuals, it confirmed that the covariance affected the estimated results of the concordance rate. The conventional concordance methods were ineffective adequately in using information within individuals. We have shown that the proposed method had a high diagnostic performance by using individual covariance. In addition, through the real example using sbp data, we confirmed the superiority of the proposed method to facilitate diagnosability by the AUC values. While we have provided only the results of the numerical simulations and a real example for the case of time point *T*=2 in this study, this proposed concordance rate can be calculated as a case of any *T*. Therefore, researching further properties of the proposed method requires simulations for the case of *T*>2.

In the proposed method, we assumed that these data are distributed as a multivariate normal distribution. For actual use in clinical settings, the concordance rate is used along with the Bland–Altman analysis to evaluate the equivalence of two measurement methods. The Bland–Altman analysis assumes normal distribution (e.g., Bland and Altman, [6]; Zou, [21]). Therefore, the assumption of the proposed method is consistent with that of the Bland–Altman analysis.

Finally, we outline the scope of four more points of future work to expand this study. First, there are no absolute criteria for the values of the proposed concordance rate, same as the conventional concordance rate. Although various criteria have been proposed, there are no common acceptable criteria for the conventional concordance rate (e.g., Saugel et al., [18]). Therefore, it is difficult to determine if the result is good, acceptable, or poor. Second, the results of the proposed concordance rate may also face problems at the time intervals between the measurement values, similar to the conventional concordance rate (e.g., Saugel et al., [18]). Therefore, the relationship between the results and length of time intervals needs to be studied further. Third, the criteria for setting the parameters of the exclusion zone have to be determined (e.g., Critchley et al., [13]). The shape of the exclusion zone may also be considered as well, for the exclusion zone is described as a rectangle such that the center of gravity is zero, other shapes should be considered as well. Fourth, while the Bland–Altman analysis is sometimes used in confirmatory clinical trials based on the statistical inference (e.g., Asamoto et al., [3]), our proposed concordance rate for the four-quadrant plot has not been established yet in this regard. Thus, concordance rate needs to be developed that also reflects statistical inference.

## Conclusion

We found that the conventional concordance rate was not a proper indicator in repeated measurements. We proposed the four-quadrant plot and its concordance rate which take into account the influence of repeated measurements within each subject. The proposed concordance rate can enhance accuracy through a calculation that depends on the numbers of agreement. The numerical simulation and the application results showed that the proposed concordance rate had more accuracy and higher diagnosability than the conventional concordance rate in *T*=2. As the proposed concordance rate provides the trending agreement from various perspectives, this new method is expected to contribute to clinical decisions in exploratory analysis. Further consideration is thus required from these points of view.

## Data Availability

The blood pressure data of package MethComp in R software. https://cran.r-project.org/web/packages/MethComp/index.html.

## Abbreviations

CO: Cardiac output
CI: Cardiac index
CCR: Conventional concordance rate
SA: Set of agreement pairs of each difference between the values of the gold standard and the experimental technique
EZ(a): The set of pairs plotted in the exclusion zone
AEz(a): The set of the “agreement” pairsin the exclusion zone
ROC: Receiver operating characteristic
AUC: Area under the curve
